# Police Perfection: Examining the Effect of Trait Maximization on Police Decision-Making

**DOI:** 10.3389/fpsyg.2020.01817

**Published:** 2020-07-22

**Authors:** Neil Shortland, Lisa Thompson, Laurence Alison

**Affiliations:** ^1^School of Criminology and Justice Studies, University of Massachusetts Lowell, Lowell, MA, United States; ^2^Centre for Critical and Major Incident Psychology, University of Liverpool, Liverpool, United Kingdom

**Keywords:** maximization, decision-making, uncertainty, individual differences, police decision-making

## Abstract

Police officers around the world must often select between equally unappealing, uncertain courses of action in an attempt to achieve the best outcome. Despite the immense importance of such decisions, there remains a lack of understanding in the study of individual differences in police decision-making. Here, using a sample of senior police officers recruited from decision-making training events across the United Kingdom (*n* = 96), we used the Least-worst Uncertain Choice Inventory For Emergency Responses (LUCIFER) to measure the effect of maximization on both domain-specific (police) and domain-general (military) decisions. In line with a wealth of research on traditional “consumer” decisions, we found that police officers who were “maximizers” found decisions more difficult. Gender and previous military experience also influenced the process of decision-making. Specifically, police officers with military experience took more time to assess the situation but were faster to choose a course of action and commit to it. Female police officers also were slower to assess the situation. As recent events show, the outcomes of police decisions have significant consequences for the public, the officers involved, the police force as a whole, and the wider population, yet psychological research has yet to fully explore the role of individual differences in how such decisions are made. While this study does not seek to identify factors associated with “good” or “better” decision-makers, it provides strong support for the need to factor in perspectives of the individual when creating theory, or applied tools, in support of police decision-making.

## Introduction

“I’m being honest with you here. I was thinking, “Hey, shit. Frankly, I don”t want to be here,” and for a fleeting moment I just wanted to get the hell out of there. I remember thinking, “There”s something wrong with this guy. I want to get the hell out of here.” He’s coming at us with this weird sort of gait and these black eyes, and there’s something wrong with him. He’s not listening to us. Let’s get the hell out of here, but I knew I couldn’t. So, for a fleeting moment we kind of retreat. I realized that, you know, you can’t really run away. This is your job. You’re going to have to handle it, but I would rather not have been there. Unfortunately, that is my job, and I remember having to tell myself, “Susan, this is your job. You have to handle this”.”

The above quote, collected by Oberweis and Musheno (1999, p. 908), presents the realities of police decision-making. Here, a potentially simple interaction manifests in a critical least-worst decision because of uncertainty about the individual they are dealing with, the motives of the individual, and the potential outcomes of this interaction. At the time of writing (June 2020), the importance of understanding the decision-making that occurs in these kinds of high-uncertainty situations cannot be underestimated. In the past few weeks alone, we have seen the aftermath of the several isolated incidents of police decision-making result in worldwide protests, calls to defund (and even disband) the police, and a new executive order signed by President Donald Trump focused on police reform. Much of the debate has focused on the concept of “bad apples” vs., “bad barrels”; i.e., the idea that “bad” police decision-making stems from “bad” individuals and/or a “bad” culture that encourages certain types of decisions (see Tator et al., 2006). While the goal of this article is not to identify the antecedents of “bad apples,” there is a very pressing need to understand the role of individual differences in police decision-making. Such knowledge is critical to inform effective theory, training, recruitment methods, and in those instances in which a police decision has a negative outcome, proper assessment of the nature of the decision, and who, or what, ultimately is to blame.

Psychologists who study decision-making in high-stakes situations (military operations, counter-terrorism operations, and emergency response situations) have reported that in many real-world decisions, the decision-maker is presented with equally unappealing options, concurrent with a *need* to choose between them (van den Heuvel et al., 2012; Alison et al., 2015; Shortland et al., 2019). In naturalistic decision-making, based on findings from research on decisions in real-life critical incidents, researchers have called these types of decisions “least-worst” (see Power and Alison, 2017a, b). Least-worst decisions are those in which every course of action is high-risk and could (potentially) have negative consequences. Further, and even more demanding, is the fact that the decision-maker considers that all anticipated outcomes appear equally aversive (or that choosing between the least-worst quickly is very difficult). Examples of least-worst decisions range from emergency responses such as the California Wildfire response, or Grenfell Tower in London, to international crises such as the Syrian Civil war (Alison et al., 2018). What all of these decisions have in common is the lack of “good” options, a need to make a decision within a given (and often short) timeframe, and significant personal, political, and societal consequences.

Naturalistic research – research which involves the observation of live decision-making (Klein, 1998) - has taken two forms. Firstly, researchers have observed the nature and occurrence of least-worst within real (or simulated) decision-making situations such as counter-terrorist operations, or responses to natural disasters (Alison et al., 2013b, 2015, 2017; van den Heuvel et al., 2014; Power and Alison, 2017a, b). Secondly, researchers have interviewed decision-makers about the process that they went through when making a least-worst decision. This work has identified the exogenous and endogenous sources of uncertainty that can influence the least-worst decision-making process (van den Heuvel et al., 2014; Shortland et al., 2019). This latter method has also allowed researchers to propose theories of how least-worst decisions are made, such as the role of competing goals/priorities and/or sacred values (Power and Alison, 2019; Shortland and Alison, 2020). While each of these strands of research has been fruitful for the study of decision-making as it occurs in the real-world, both lack the ability to explore the role of individual differences in least-worst decision-making. Even research in wider fields that make similar least-worst choices (such as the emergency room, medical fields, or emergency responses) has often focused on exploring the psychological process of making decisions and not the individual personality factors that govern individual differences in how well people make these decisions (e.g., Shaban, 2015). This leaves a significant gap in our understanding of least-worst decision-making given that the same naturalistic research has noted that some individuals and groups are better able to commit to least-worst choices than others (Shortland et al., 2019).

### Police Decision-Making

Much of the existing literature on police decision-making focuses primarily on decisions made throughout the investigative process, including what criminal investigations to prioritize and what strategies to use during interrogations and other inquiries into criminal cases (Ask and Alison, 2017). While studying the investigative process is an essential aspect of understanding police decision-making, these investigations often take place over a longer stretch of time, thus allowing more information to be gathered prior to making a decision (e.g., an arrest), when compared to a critical incident (e.g., a terrorist attack), in which less time can be allocated for law enforcement to gather all of the details before taking action. In these different types of situations, the amount of time available impacts whether risk assessments can effectively be made using an analytical versus intuitive approach. Other police decision-making research includes the effects of race, gender, and age on decision-making patterns during traffic stops (Schafer et al., 2006), or how extralegal factors impact decision-making policies for when to make an arrest for spousal abuse (Waaland and Keeley, 1985; Kane, 1999).

In a study that applied a naturalistic decision-making paradigm when studying use-of-force during encounters with civilians, Hine et al. (2018) found that officers’ decision-making was more aligned with an intuitive style (i.e., automatic, unconscious decision-making, and heuristics), although an analytical approach was used to conduct mental simulations of possible outcomes. Furthermore, officers also experienced various cognitive, perceptual, and physiological impairments that affected their ability to successfully use force techniques, thus increasing the potential for risk of injury to either the officer or suspect (Hine et al., 2018).

Prior research has also shown that police officers managed uncertainty in dynamic, high-risk situations by seeking out additional information and updating their assessments of a given situation based on their previous experience, as a way to reduce the levels of uncertainty experienced during three phases of the decision-making process: situation assessment, plan formulation, and plan execution. In the event that uncertainty persisted to the time when a plan would be executed, it would be further “reduced” by either relying on standard operating procedures or by purposefully deferring the execution of a plan while also preparing for potential “worst-case scenarios” (van den Heuvel et al., 2014).

Other work has used a naturalistic decision-making approach to study police decision-making during simulations of major events, such as political or sporting events (Pais and Felgueiras, 2016). Faced with time pressure, as well as incomplete knowledge and limited capability to process information, effective decision-makers tended to *satisfice* and focused on attaining acceptable solutions for managing traffic control and monitoring operations (Pais and Felgueiras, 2016). Two recent studies have elucidated the important role that individual differences in personality traits associated with decision-making may play in police decision-making. First, Alison et al. (2013a) used a series of simulated rape cases to examine the effects of (a) internal time urgency, (b) experience, and (c) fluid mental ability on diagnostic hypotheses and an officer’s ability to prioritize information. In an experimental simulation in which half of the subjects were subjected to a time pressure manipulation (although both groups had equal amounts of time), the subjective perception of having less time caused participants to generate a reduced number of hypotheses. What is more important is that this effect was moderated by individual differences in time urgency. Specifically, individuals who tended to perceive time to pass more slowly (low time urgency) continued to generate hypotheses despite the presence of time pressure. Time pressure also increased action prioritization, but only in those officers with low time urgency or high fluid ability. In a second study by Kim et al. (2020), Korean detectives participated in a series of simulated investigative scenarios to investigate the extent to which individual differences in (a) domain-specific experience, (b) fluid intelligence, (c) need for closure (NFC), and (d) time urgency moderated the effect of time pressure on investigative hypothesis generation. Here, time pressure directly decreased the quantity and quality of hypotheses generated and, again, low time urgency moderated the effect of time pressure on the number and quality of hypotheses generated. Low NFC also moderated the impact of time pressure on the number of hypotheses generated. These studies together reinforce the importance role that individual differences in traits associated with decision-making can play on police decision-making during operationally relevant tasks.

### Maximization

Recent work with members of the military has emphasized the importance of individual differences in trait maximization in least-worst decisions because they require the individual to satisfice in choosing the least-worst option (Shortland et al., 2020). Maximization moderates the “paradox of choice” in that when people are attracted to a larger number of alternatives they are often more dissatisfied with their eventual choice (Dar-Nimrod et al., 2009; see also Schwartz, 2004). Individual differences in maximization reflect peoples’ tendencies to seek out the “best” possible option vs., satisficing for an option that is “good enough,” according to their personal standard (Schwartz, 2004). Maximization has been extensively studied by psychologists, and differences in decision-making strategies and decision-making outcomes have been found between maximizers (the term for those who score high on scales of maximization) and satisficers (the term for those who score low on maximization). Maximizers are more likely to procrastinate (Osiurak et al., 2015) and to engage in counterfactual thinking and “what if” thoughts about decisions (Roese, 1994; Kahneman, 1995; Schwartz et al., 2002). Maximizers also prefer different types of decisions (i.e., those with high numbers of possible alternatives) and are more likely to adopt decision-making strategies that reflect rational-cognitive models (Cheeks and Schwartz, 2016). Thus, the past wealth of research reinforces that maximization is a key psychological variable when examining individual differences in decision-making (see Cheeks and Schwartz, 2016).

Maximizing is not only associated with the process of decision-making, but also the outcomes. For example, maximizers have lower overall self-esteem (Schwartz et al., 2002), maximizers report lower levels of happiness (Schwartz et al., 2002; Polman, 2010) and also lower life satisfaction (Schwartz et al., 2002; Dahling and Thompson, 2012). Post-decision, maximizers are more prone to express regret (Parker et al., 2007; Moyano-Díaz et al., 2013; Besharat et al., 2014), and report that they are more of a “perfectionist” (Schwartz et al., 2002; Bergman et al., 2007; Chang et al., 2011; Dahling and Thompson, 2012). Maximizers are also less optimistic (Schwartz et al., 2002); maximizers report that they are greedier (Seuntjens et al., 2015) and that they are more neurotic than those who do not consistently seek to maximize a given decision (Schwartz et al., 2002; Purvis et al., 2011).

Maximization studies continually focus on decisions pertaining to consumer goods (e.g., Diab et al., 2008; Weaver et al., 2015; Kokkoris, 2018), yet from an applied perspective, there are many benefits of integrating maximization into the study of high-uncertainty least-worst decisions. First and foremost, least-worst decision-making is often shown to become de-railed, resulting in decision inertia. Decision inertia involves the delaying of the decision-making process through either avoidance, redundant deliberation, or failure to implement a decision (Alison et al., 2018). In many applied situations the failure to make a decision in time (or at all), is as damaging, if not worse, than an incorrect decision (Shortland et al., 2019). From a theoretical perspective maximization may play a critical role in the emergence of decision inertia given that current theories of least-worst decision-making emphasize that decisions become de-railed through a failed commitment to making the “best” choice (van den Heuvel et al., 2012; Power and Alison, 2017b; Shortland and Alison, 2020). This puts a precedent on efforts to explore the potential role that maximization may play in applied samples and instances of applied decision-making, in support of both theoretical advances in the study of decision-making, as well as applied issues of selection, training, and recovery.

### This Study

Based on the stated importance of maximization and the lack of theoretical input into the role of individual differences in the process of police decision-making, this study utilized a recently developed measure of least-worst decision-making in high-uncertainty environments to an applied sample of police officers from the United Kingdom to explore the effect of individual differences in maximization on police decision-making. Based on a review of extant literature of maximization (see above) we hypothesize that:

H_1_: Individuals with greater maximization tendencies will find decisions to be more difficult.
H_2_: Individuals with higher levels of maximization will be slower to decide.
H_3_: Individuals with higher levels of maximization will be more likely to make choices that reflect tendencies of avoidance.

## Materials and Methods

### Participants

The sample for this study comprised of 96 senior police officers recruited from several different constabularies of the United Kingdom Police Forces (73.96% male), with an age range of 20–56 years (*M* = 40.69, *SD* = 7.81). On average officers had over 17 years’ experience serving as a police officer (*M* = 17.46). All participants were serving as active members of the United Kingdom Police Force when they completed this study.

### Materials

#### Maximization

The current study uses Turner et al.’s (2012) 34-item Maximization Inventory. Turner et al. (2012) measures three components of maximization: satisficing (10 items), decision difficulty (12 items), and alternative search (12 items). Each item is scored using a 6-point scale ranging from “Strongly Disagree” (1) to “Strongly Agree” (6). Satisficing measures the degree to which someone chooses outcomes that reach the threshold of “acceptability,” rather than ones much closer to optimal. This subscale includes items such as, “At some point you need to make a decision about things.” Decision difficulty measures the frustration—or difficulty—that one experiences when making a choice. Example items in this subscale include, “I am usually worried about making a wrong decision.” Finally, alternative search measures an individual’s tendency to seek all available options before committing to a choice. Items in this subscale include, “I take the time to consider all alternatives before making a decision.”

#### Decision-Making

The research used the LUCIFER (Least-worst Uncertain Choice Inventory for Emergency Responses; Shortland et al., 2020) decision-making measure. The following dependent variables are collected by the LUCIFER research method (both on average across the scenarios, and per scenario):

1. Situational Awareness Time (SAT): the amount of time it takes the participant to declare that they are “ready” to make a decision after they have listened to an audio inject that outlines the situation.
2. Choice time (CT): participants are required to choose between two courses of action. The amount of time it takes them to decide between A and B is recorded in LUCIFER. LUCIFER operationalizes CT by measuring the amount of time it takes a participant to make their last “click” on an option on the page (both first and last page clicks are recorded for each step of the scenario).
3. Decision Time (DT): the overall time it takes a participant to declare they are ready to “commit” to their choice (i.e., submit).
4. Commitment Time (ComT): ComT is calculated as the difference between choosing (CT) and submitting (DT). ComT was therefore calculated as ComT = DT – CT.
5. Decision Difficulty (DD): Participants complete a five-item decision difficulty scale (see Hanselmann and Tanner, 2008). This DD measure included the items, “For me this decision is…” “very easy” (1) to “very difficult” (7). Four additional items ask the participant to rate their level of agreement with statements regarding the time they needed, how certain they were and how committed they were to their choice (“strongly disagree” [1] to “strongly agree” [7]). Each item was scored using a 7-point Likert scale.
6. Approach/Avoidance (AA): each decision offered two choices. One choice was an approach outcome, in which they could actively seeking to make a positive impact on the situation. The second choice was an avoidance outcome, that allowed them to withdraw and prevent further harm (see Power, 2018). Summing the total number of approach decisions made (maximum score: 10) across the scenarios gave an overall approach/avoidance tendency (high score: approach, low score: avoid).

Based on previous research, which has shown the role of NFC in decision-making (Kim et al., 2020; Shortland et al., 2020), this study controlled for Need for Closure (NFC). NFC measures the degree to which an individual wants to obtain definitive answers and their comfort with uncertainty (Kruglanski, 1989, p. 14). NFC was measured via the five-facet scale proposed by Kruglanski et al. (1993).

### Procedure

The research team (first and third author) collected data in person at a range of training events hosted throughout the country. The study was administered to participants via Apple iPad and or a personal computer. The study was hosted on Qualtrics, and all data was collected and held on the Qualtrics server. Before completing the LUCIFER task, all participants completed the psychometric battery (maximization and NFC). Informed consent was provided digitally before beginning the study. Participants were reminded of their ability to end testing at any time and were supervised by a test proctor throughout their testing. They were asked to complete the battery in a single session.

#### Decision-Making Task (LUCIFER)

LUCIFER is a decision-making task that was developed to support on-going research into the how individuals make least-worst decisions under conditions of uncertainty (Shortland et al., 2020). LUCIFER was developed in collaboration with the Army Research Institute Foundational Science Research Unit. To date, LUCIFER has been used with a range of applied groups (soldiers, police officers, firefighters, etc.). Decision-making scenarios used in LUCIFER were developed from data collected from qualitative interviews with police officers and soldiers (see Shortland et al., 2019; Shortland and Alison, 2020). These scenarios were then condensed down to two critical decision points, which are then presented to the participant via a recorded audio feed with corresponding background noise. The individual is then presented with a 2-alternative forced choice (2AFC) which represent either an approach choice (make a positive impact on the situation), or an avoidance approach (avoid a negative impact on the situation). All audio feeds are recorded by either members of the armed forces or paid actors and provide the participant with an assessment of the situation and a required action. All scenarios have been “civilianized” and piloted with a sample of undergraduate students to ensure than they can be comprehended by those with no specialized knowledge of the situation (i.e., the scenarios contain no specialized language). After making their first decision, participants are exposed to a second inject (second-step) that either tests their commitment to the course of action they chose, or presents a further step in the scenario. Again, after being exposed to this second audio inject, the participant is asked to choose their course of action. After completing both steps, the participant records their confidence level and completes the decision difficulty questionnaire outlined above. The version of LUCIFER used in this study involves 3 Police scenarios and 2 non-Police (military) scenarios.

## Study 1

### Results

#### Overall Performance

On average, participants took 22 s to understand the situation and declare themselves “ready” to decide (*M* = 21.97, *SD* = 16.39; see Table 1). On average, participants took just over 14 s to decide (DT, *M* = 14.42, *SD* = 12.93). Committing to this decision took participants, on average, 4 s (CT, *M* = 4.52, *SD* = 4.73). On average, participants scored the scenarios as ‘medium’ difficulty (*M* = 15.04, *SD* = 3.40), and there was a slight tendency to make “Approach” choices (*M* = 4.49, *SD* = 1.53).

**TABLE 1:** Descriptive statistics for total police sample.

**Variable**	**Mean**	**Standard deviation**	**Minimum**	**Maximum**
**Outcome variable (*N* = 960)**				
Situational awareness time (SAT)	21.97	16.39	3.53	100.98
Decision time (DT)	14.42	12.93	2.58	93.28
Choice time (CT)	9.90	10.90	1.76	79.34
Commitment time (ComT)	4.52	4.73	0.00	26.16
Decision difficulty (DD)	15.04	3.40	1.70	22.81
**Individual level variables (*N* = 96)**				
Need for closure score (NFC)	48.94	9.44	29.00	73.00
Maximization score (MAX)	120.54	16.97	84.00	172.00
Avoidance score (AA)	4.49	1.53	1.00	8.00
Age	40.69	7.81	20.00	56.00
Police experience	16.56	8.19	2.00	35.00
	*N*	%		
Type (ref = police only)				
Military experience (yes = 1)	40	41.67		
Gender (ref = female)				
Male (yes = 1)	71	73.96		
**Scenario level variables (*N* = 10)**				
Military scenarios	6			

##### Maximization

Overall, participants’ Maximization scores ranged from 84.0 to 172.0 (*M* = 120.54, *SD* = 16.97). Preliminary analyses included a series of Pearson’s correlations that indicated that Maximization was positively correlated with decision difficulty (*r* = 0.249, *n* = 96, *p* = 0.014), Need for Closure (*r* = 0.339, *n* = 96, *p* = 0.001), and age (*r* = 0.249, *n* = 96, *p* = 0.014). All other correlations were not statistically significant.

#### Total Police Sample

Multi-level modeling (MLM) tested the effect of Maximization on decision-making while controlling for NFC and other variables and nesting decision-making across scenarios by the individual participant. 5 scenarios, with ten total decisions, and 96 participants resulted in a total of 960 data points per dependent variable. Multi-level modeling for each of the dependent variables thus organized the total 960 data points both by the random effects of the five scenarios (ten decisions) and by the 96 participants. Using this structure, a two-level MLM was used to estimate the main effect of Maximization on SAT, DT, CT, ComT, DD, and AA (see Table 2), controlling for Need for Closure, scenario type, military experience, age, and gender.

**TABLE 2:** Multilevel linear regressions for preference and difficulty scorings with crossed-random effects for total police sample.

**Models**	**β**	***SE***	**Odds ratio [exp(β)]**	***p***
**(1) Situational awareness (*N* = 960)**				
Constant	14.859	12.612	2.840*10^6^	0.239
Need for closure score (NFC)	0.218	0.202	1.243	0.281
Maximization score (MAX)	0.010	0.113	1.010	0.931
Military scenario (yes = 1)	6.584	3.669	723.556	0.073
Type (ref = police only)				
Military experience (yes = 1)	12.456	4.218	2.568*10^5^	0.003**
Gender (male = 1)	–8.517	4.034	2.001*104-	0.035*
Age	–0.131	0.391	0.877	0.738
Police experience (years)	0.550	0.382	1.734	0.149
**(2) Choice time (*N* = 960)**				
Constant	6.138	6.841	462.902	0.370
Need for closure score (NFC)	–0.039	0.108	0.962	0.718
Maximization score (MAX)	0.023	0.060	1.023	0.703
Military scenario (yes = 1)	1.724	2.649	5.605	0.515
Type (ref = police only)				
Military experience (yes = 1)	–6.010	2.247	0.002	0.007**
Gender (male = 1)	–1.260	2.149	0.284	0.557
Age	0.221	0.208	1.247	0.290
Police experience (years)	–0.205	0.203	0.814	0.312
**(3) Decision time (*N* = 960)**				
Constant	10.146	8.575	2.550*10^4^	0.237
Need for closure score (NFC)	–0.098	0.125	0.906	0.431
Maximization score (MAX)	0.073	0.070	1.076	0.296
Military scenario (yes = 1)	1.795	5.157	6.018	0.728
Type (ref = police only)				
Military experience (yes = 1)	–10.011	2.610	4.492*105-	0.000***
Gender (male = 1)	–0.256	2.496	0.774	0.918
Age	0.307	0.242	1.360	0.204
Police experience (years)	–0.335	0.236	0.715	0.156
**(4) Commitment time (*N* = 960)**				
Constant	4.009	4.151	55.079	0.334
Need for closure score (NFC)	–0.060	0.059	0.942	0.309
Maximization score (MAX)	0.050	0.033	1.051	0.127
Military scenario (yes = 1)	0.071	2.740	1.074	0.979
Type (ref = police only)				
Military experience (yes = 1)	–4.001	1.224	0.018	0.001***
Gender (male = 1)	1.005	1.171	2.731	0.391
Age	0.087	0.114	1.091	0.444
Police experience (years)	–0.130	0.111	0.878	0.242
**(5) Decision difficulty score (*N* = 960)**				
Constant	10.719	2.719	4.520*10^4^	0.000
Need for closure score (NFC)	–0.039	0.044	0.962	0.379
Maximization score (MAX)	0.070	0.025	1.072	0.005**
Military scenario (yes = 1)	1.445	0.428	4.240	0.001***
Type (ref = police only)				
Military experience (yes = 1)	–0.486	0.925	0.615	0.599
Gender (male = 1)	–0.658	0.884	0.518	0.457
Age	0.157	0.086	1.170	0.068
Police experience (years)	–0.136	0.084	0.873	0.103
**(6) Avoidance score (*N* = 960)**				
Constant	4.973	0.319	144.489	0.000
Need for closure score (NFC)	0.008	0.006	1.008	0.191
Maximization score (MAX)	–0.001	0.004	0.999	0.763
Military scenario (yes = 1)	6.717*1015-	3.888*108-	1.000	0.999
Type (ref = police only)				
Military experience (yes = 1)	0.015	0.132	1.015	0.908
Gender (male = 1)	–0.101	0.128	0.904	0.433
Age	–0.006	0.010	0.994	0.540
Police experience (years)	–0.008	0.011	0.992	0.427

*p < 0.05, **p < 0.01, ***p < 0.001. A series of models were also run examining interaction effects between Maximization scores and scenario type on each of the outcome variables, but there was no evidence to suggest that there was a significant interaction.

Overall, across these models, “maximizers” (those who score high on trait maximization) appear to perceive LUCIFER decisions as more difficult, with an average increase of 0.070 points in difficulty scores for every one-point increase in maximization score (*p* = 0.005). The type of scenario also affected decision difficulty, with the odds of perceived difficulty being higher for military scenarios compared to non-military scenarios (OR = 4.240, *p* = 0.001).

Situational awareness time was positively associated with having military experience, with officers with military experience taking longer to assess a situation on average (OR = 2.568^∗^10^5^, *p* = 0.003). Males also tended to take less time to assess a situation (OR = 2.001^∗^10^–4^, *p* = 0.035). On average, police officers with military experience tended to have faster choice times than officers with no military experience (OR = 0.002, *p* = 0.007). Decision time was positively associated with having military experience, with officers with military experience taking longer to make a decision on average (OR = 4.492^∗^10^–5^, *p* < 0.001). On average, police officers with military experience tended to take less time to commit to a choice than officers with no military experience (OR = 0.018, *p* = 0.001). Tendency to avoid did not appear to be significantly associated with any of the model variables.

Within the total sample, we fail to reject our first hypothesis, with higher maximization scores positively associated with increased decision difficulty within a given scenario. There did not appear to be any empirical support for our second or third hypotheses, with maximization having no statistically significant impact on either reaction times or tendency to avoid.

#### Discussion

Within the total sample, we fail to reject our first hypothesis, with higher maximization scores positively associated with increased decision difficulty within a given scenario. There did not appear to be any empirical support for our second or third hypotheses, with maximization having no statistically significant impact on either reaction times or tendency to avoid. While the full discussion of these results will occur in the proceeding overall discussion section, there is one finding that warrants further attention and exploration: the role of participant membership in the military. In this study, those who served in the military alongside the police force were overall slower to assess the situation, but faster to decide than those who had no military experience. To date, there has been no investigation of the role of having military experience on police decision-making. Such work is especially prudent given that emerging work on least-worst decision-making has argued that military personnel are less prone to redundant deliberation (a form of indecision) in the face of least-worst decisions (see Shortland et al., 2019; Shortland and Alison, 2020). That said, this hypothesis has not been experimentally tested. Thus, below we offer a preliminary test of the role of military experience on police decision-making (using data from the sample collected for Study 1).

## Study 2

### Results

#### Group Differences

When comparing between police officers who had served as military personnel and those who had military experience took, on average, 6.89 s longer to assess the situation, *t*(79.190) = −2.034, *p* = 0.045, but 5.68 s less time to make a decision, *t*(70.563) = 2.073, *p* = 0.042, and 2.50 s less time to commit to their choice, *t*(93.997) = 2.788, *p* = 0.006. There were no significant group differences in maximization scores, perceived decision difficulty, need for closure, tendency to avoid, or age. However, there was a significant group difference in years of police experience, with police only samples reporting an average of 8.85 years more experience in law enforcement than the hybrid police/military sample, *t*(60.972) = 5.666, *p* < 0.001. For gender, males were distributed evenly between those with military experience and those without, while only 20% of female participants reported any military experience (see Table 3).

**TABLE 3:** Group differences for total sample.

	**Total sample (*N* = 96)**	**Police only sample (*n* = 56)**	**Police/military sample (*n* = 40)**	**Mean difference**
**SAT** (seconds)	21.97 (16.39)	19.10 (15.47)	25.99 (16.98)	6.89 *
**DT** (seconds)	14.42 (12.93)	16.78 (11.25)	11.10 (14.48)	5.68 *
**CT** (seconds)	9.90 (10.90)	11.22 (9.75)	8.05 (12.21)	3.17
**ComT** (seconds)	4.52 (4.73)	5.56 (5.15)	3.06 (3.64)	2.50 **
**DD**	15.04 (3.40)	15.07 (2.69)	15.00 (4.24)	0.07
**NFC**	48.94 (9.44)	49.54 (9.24)	48.10 (9.77)	1.44
**Max**	120.54 (16.97)	121.55 (18.61)	119.12 (14.46)	2.43
**Avoid**	4.49 (1.53)	4.48 (1.69)	4.50 (1.28)	0.02
**Age**	40.69 (7.81)	42.00 (6.70)	38.90 (8.91)	3.10
**Police experience**	16.56 (8.19)	20.39 (5.45)	11.54 (8.52)	8.854 ***
**Gender:**				
**Male**	71	36	35	*
**Female**	25	20	5	

*p < 0.05, **p < 0.01, ***p < 0.001.

#### Police Only Sample

Across these different models, as presented in Table 4, maximization appears to have a positive association with perceived decision difficulty, with an average increase of 0.099 points in difficulty scores for every one-point increase in maximization score (*p* < 0.001); scenario type also affected decision difficulty, with the odds of perceived difficulty being higher for military scenarios compared to non-military scenarios (OR = 2.438, *p* = 0.029).

**TABLE 4:** Multilevel linear regressions for preference and difficulty scorings with crossed-random effects for police only sample.

**Models**	**β**	***SE***	**Odds ratio [exp(β)]**	***p***
**(1) Situational awareness (*N* = 560)**				
Constant	4.688	25.970	108.603	0.857
Need for closure score (NFC)	–0.023	0.305	0.978	0.941
Maximization score (MAX)	0.023	0.146	1.023	0.876
Military scenario (yes = 1)	2.977	3.124	19.633	0.341
Gender (male = 1)	–10.142	4.595	3.940*105-	0.027*
Age	–0.081	0.820	0.922	0.922
Police experience (years)	1.109	0.728	3.033	0.128
**(2) Choice time (*N* = 560)**				
Constant	11.561	18.407	1.049*10^5^	0.530
Need for closure score (NFC)	–0.138	0.214	0.871	0.518
Maximization score (MAX)	0.016	0.102	1.016	0.877
Military scenario (yes = 1)	3.612	4.445	37.033	0.416
Gender (male = 1)	–1.133	3.219	0.322	0.725
Age	0.208	0.574	1.231	0.718
Police experience (years)	–0.507	0.510	0.602	0.320
**(3) Decision time (*N* = 560)**				
Constant	20.646	21.063	9.253*108	0.327
Need for closure score (NFC)	–0.265	0.238	0.767	0.265
Maximization score (MAX)	0.047	0.114	1.048	0.677
Military scenario (yes = 1)	3.894	8.175	49.098	0.634
Gender (male = 1)	–0.194	3.575	0.824	0.957
Age	0.256	0.638	1.292	0.688
Police experience (years)	–0.811	0.566	4.443	0.152
**(4) Commitment time (*N* = 560)**				
Constant	9.085	9.638	8818.050	0.346
Need for closure score (NFC)	–0.127	0.108	0.881	0.241
Maximization score (MAX)	0.032	0.052	1.032	0.541
Military scenario (yes = 1)	0.282	3.962	1.326	0.943
Gender (male = 1)	0.939	1.625	2.557	0.563
Age	0.048	0.290	1.050	0.867
Police experience (years)	–0.304	0.257	0.738	0.237
**(5) Decision difficulty score (*N* = 560)**				
Constant	15.157	4.244	3.825*10^6^	0.000
Need for closure score (NFC)	–0.072	0.050	0.931	0.150
Maximization score (MAX)	0.099	0.024	1.104	0.000***
Military scenario (yes = 1)	0.891	0.407	2.438	0.029*
Gender (male = 1)	–0.108	0.752	0.898	0.886
Age	0.010	0.134	1.101	0.940
Police experience (years)	–0.038	0.119	0.963	0.752
**(6) Avoidance score (*N* = 560)**				
Constant	6.311	0.825	550.351	0.000
Need for closure score (NFC)	–0.019	0.011	0.981	0.087
Maximization score (MAX)	0.004	0.005	1.004	0.472
Military scenario (yes = 1)	9.053*1015-	1.018*107-	1.000	0.999
Gender (male = 1)	–0.206	0.164	0.814	0.210
Age	0.003	0.026	1.003	0.907
Police experience (years)	–0.090	0.025	0.914	0.000***

*p < 0.05, ***p < 0.001. A series of models were also run examining interaction effects between Maximization scores and scenario type on each of the outcome variables, but there was no evidence to suggest that there was a significant interaction.

Situational awareness time was significantly associated with gender, and males tended to take less time to assess a situation (OR = 3.940^∗^10^–5^, *p* = 0.027). No other reaction time outcomes appeared to be associated with any of the model variables. Tendency to avoid was negatively associated with police experience, with every year of experience leading to an average decrease of 0.090 points in avoidance score.

Within the police only sample, we fail to reject our first hypothesis, with higher maximization scores positively associated with increased decision difficulty within a given scenario. There did not appear to be any empirical support for our second or third hypotheses, with maximization having no statistically significant impact on either reaction times or tendency to avoid. These findings are consistent with the findings for the total sample used in the study.

#### Police/Military Hybrid Sample

Across these different models (see Table 5), maximization appears to have a negative association with tendency to avoid, with an average decrease of 0.027 points in avoidance scores for every one-point increase in maximization score (*p* < 0.001). Tendency to avoid was also positively associated with NFC, with an average increase in avoidance score of 0.032 points for every one-point increase in NFC (*p* < 0.001). Likewise, tendency to avoid was positively associated with police experience, with an average increase in avoidance score of 0.018 points for every extra year of experience (*p* = 0.030). Additionally, tendency to avoid was affected by gender, with males being more likely to have a higher avoidance score than females (OR = 2.601, *p* < 0.001).

**TABLE 5:** Multilevel linear regressions for preference and difficulty scorings with crossed-random effects for police/military hybrid sample.

**Models**	**β**	***SE***	**Odds ratio [exp(β)]**	***p***
**(1) Situational awareness (*N* = 400)**				
Constant	19.698	14.700	3.588*10^8^	0.180
Need for closure score (NFC)	0.586	0.270	1.798	0.030*
Maximization score (MAX)	0.184	0.180	1.202	0.306
Military scenario (yes = 1)	11.208	5.741	7.375	0.051
Gender (male = 1)	–3.689	7.634	0.025	0.629
Age	0.086	0.436	1.089	0.844
Police experience (years)	–0.128	0.456	0.880	0.779
**(2) Choice time (*N* = 400)**				
Constant	0.217	3.924	1.242	0.956
Need for closure score (NFC)	–0.006	0.073	0.994	0.939
Maximization score (MAX)	–0.007	0.049	0.993	0.885
Military scenario (yes = 1)	–0.697	1.310	0.498	0.595
Gender (male = 1)	–0.542	2.062	0.582	0.793
Age	0.198	0.118	1.219	0.092
Police experience (years)	–0.066	0.123	0.936	0.590
**(3) Decision time (*N* = 400)**				
Constant	–1.016	5.585	0.362	0.856
Need for closure score (NFC)	–0.036	0.104	0.965	0.731
Maximization score (MAX)	0.049	0.069	1.050	0.478
Military scenario (yes = 1)	–0.896	1.881	0.408	0.634
Gender (male = 1)	1.336	2.927	3.802	0.648
Age	0.284	0.167	1.329	0.089
Police experience (years)	–0.138	0.175	0.871	0.428
**(4) Commitment time (*N* = 400)**				
Constant	–1.223	3.193	0.291	0.699
Need for closure score (NFC)	–0.030	0.058	0.970	0.605
Maximization score (MAX)	0.056	0.039	1.058	0.149
Military scenario (yes = 1)	–0.199	1.260	0.819	0.874
Gender (male = 1)	1.877	1.647	6.535	0.255
Age	0.086	0.094	1.090	0.361
Police experience (years)	–0.072	0.098	0.931	0.464
**(5) Decision difficulty score (*N* = 400)**				
Constant	8.904	4.145	7363.768	0.032
Need for closure score (NFC)	–0.008	0.079	0.992	0.918
Maximization score (MAX)	0.024	0.053	1.024	0.649
Military scenario (yes = 1)	2.154	0.532	8.622	0.000***
Gender (male = 1)	–2.021	2.231	0.133	0.365
Age	0.214	0.127	1.238	0.093
Police experience (years)	–0.151	0.133	0.860	0.257
**(6) Avoidance score (*N* = 390)^a^**				
Constant	3.454	0.164	31.625	0.000
Need for closure score (NFC)	0.032	0.008	1.033	0.000***
Maximization score (MAX)	–0.027	0.005	0.973	0.000***
Military scenario (yes = 1)	-5.854*1015-	1.231*10^8^	1.000	0.999
Gender (male = 1)	0.956	0.181	2.601	0.000***
Police experience (years)	0.018	0.008	1.018	0.030*

*p < 0.05; ***p < 0.001. A series of models were also run examining interaction effects between Maximization scores and scenario type on each of the outcome variables, but there was no evidence to suggest that there was a significant interaction. ^*a*^“Age” was removed as a model variable due to multicollinearity issues with “Police Experience” in the avoidance score model.

Situational awareness time was positively associated with need for closure, with an average increase in SAT of 0.586 s for every one-point increase in NFC (*p* = 0.030). Scenario type affected decision difficulty, with the odds of perceived difficulty being higher for military scenarios compared to non-military scenarios (OR = 8.622, *p* < 0.001). Neither choice time, decision time, nor commitment time appeared to be associated with any of the model variables.

Within the hybrid sample of individuals with experience in both law enforcement and military service, we reject our first and second hypotheses, with higher maximization scores having no statistically significant impact on either decision difficulty or reaction times. However, unlike the total sample or police only subsample, we fail to reject our third hypothesis, with individuals with higher maximization scores showing less of a tendency to avoid.

## Discussion

In terms of the psychology of decision-making, there is an urgent need to understand how people make decisions in the face of high uncertainty. At the time of writing, political leaders, CEOs, medical staff, and police officers are making a range of decisions in the face of the unprecedented COVID-19 global pandemic, and the decision-making of police officers in the line of duty is coming under increased scrutiny. Several recent cases of police decision-making have resulted in global protests, riots, the disbanding of some police departments, and executive orders focused on police reform. Now, more than ever, psychologists need to begin to understand the realities of police decision-making and the processes that underpin the ability of police officers, in the face of immense strain and uncertainty, to navigate non-ideal options and commit to a course of action. To date, the majority of research on police decision-making has focused on prioritization of certain criminal cases and the employment of interrogation techniques (Ask and Alison, 2017), the effects of salient demographic markers (i.e., age, race, and gender) on decision-making patterns during traffic stops (Schafer et al., 2006), or how external factors impact decision-making policies for domestic violence arrests (Waaland and Keeley, 1985; Kane, 1999). However, several recent studies have emphasized the potential importance of factoring individual differences in personality variables associated with decision-making into our analyses (Alison et al., 2013b; Kim et al., 2020). In this study, we sought to extend the study of individual differences in police decision-making by examining the effect of individual differences in trait maximization on a range of police-related (and non-police related) decisions. In doing so, we identified several interesting tendencies associated with both personality, and experience related traits. First and foremost, confirming our first hypothesis, police officers who were maximizers found decisions harder, though their decision-making speed was unaffected. However, this study also highlighted important decision-making differences between those who had military experience and those who did not. Specifically, those senior officers with military experience were slower to assess the situation, but faster to decide. Male officers were also faster to assess the situation than females. These findings show the importance of considering experiential and personality based individual differences in how police officers make decisions under conditions of high-uncertainty and least-worst options.

Maximization is the individual tendency to “maximize” outcomes by seeking the best possible choice, rather than settling for a “acceptable” choice (for reviews, see Cheeks and Schwartz, 2016; Misuraca and Fasolo, 2018), and the effect of maximization on decision-making styles and outcomes has been studied by psychologists for the past several decades. In line with this previous research, this study hypothesized that individual differences in trait maximization would influence police officers when making high-uncertainty decisions. The types of decisions that police officers face place an immense strain on the person, and indeed our own psychological theories of how decisions are made. While the majority of naturalistic research has focused on the processes through which these decisions are made (e.g., Alison et al., 2013a), what is often missing is a focus on the individual traits that may explain how and why people show differences in their ability to make effective decisions in these situations. This is why maximization is so important because it provides a metric of the observable tendency that people have to try and make the best of a bad situation (Shortland et al., 2019). In line with our hypotheses police officers with high trait maximization found decisions harder. They did not, however, take to comprehend the situation and be “ready” to decide, take longer to select a course of action (when presented with a binary A or B choice), or take longer to commit to a course of action. This supports the notion that maximizers find decisions more difficult (Kim and Miller, 2017), though we did not find that maximizers were slower, or more inclined to make avoidant choices (Parker et al., 2007). It is especially interesting to see that these findings extend into a sample of police officers who have face such decisions frequently (Power, 2018). This shows the potential theoretical and practical utility of maximization. It is also important to consider why some of our hypotheses were rejected by this research; namely that maximizers should be slower and more prone to avoidant decisions. One reason for this may be the nature of the group, and how this sample differs from the “usual” sample of research on maximization. Police officers are subjected to rigorous training, especially in decision-making, and it is very viable to propose that decision-making and/or wider training experienced as a police officer alters the natural effect of maximization on the process of making a decision under conditions of uncertainty. It is viable thus that with training the conscious tendency to seek alternatives could be overridden in situations in which there is time pressure and a need to act. Future work manipulating time pressure (e.g., Kim et al., 2020) should explore this.

This work also identified differences between those police officers who have military experience and those who do not. This finding is in accordance with an oft-observed comment by those who study decision-making under uncertainty that there are differences between those who work in emergency services and those who operate within the military (Shortland et al., 2019). Through a series of interviews with members of the Armed Forces, previous research suggests that military personnel, when compared to non-military personnel, are generally more resistant to decision inertia and better able to commit to least-worst choice under uncertainty (Shortland et al., 2019). To be clear, in this study we are not saying that police officers with military experience make “better” decisions, just that in this study they were slower to assess the situation, but once they had assessed the situation they were universally faster throughout the remaining stages of the decision-making process. This finding implies that officers with military experience may process decisions differently and this could, in turn, make them better equipped to make certain *types* of decisions in certain *types* of environment. Despite the potential limitations of the comparison here (differences in overall experience, small sample size), what this work does support is the potential utility of focusing on the psychological differences between police officers who have military experience in terms of performance. This is a highly relevant issue within Industrial/Organizational psychology in which we would look to the matching of people to tasks. A wealth of future research is needed in this area, but it is warranted to propose that police officers with military experience may be suited to certain types of tasks depending on the nature of the decisions they are facing. The nature of decisions that police officers face are immensely diverse, ranging from slow-bun investigative decisions, to sudden shoot/don’t shot decisions. This research implies that significant psychological support can be provided by factoring in the interaction of individual differences in decision-making process and the nature of the decisions that the individual is likely to face in the field.

### Limitations

Despite the potential utility of these findings, it is important to consider the limitations of this study. First and foremost, this is a small, and selective sample of police officers who are not representative of the police forces as a whole, nor may their culture transition globally. For example, authors are increasingly commenting on the “unique” aspects of police culture in certain countries (such as the United States; see Demirkol and Nalla, 2017). These findings should thus be treated with caution, and indeed replicated cross-culturally and indeed within different countries and across forces to examine the boundaries of maximization (as well as identify unique aspects that may mitigate the effect of this trait). That said, a recent study also found that maximization also affected military decision-making with a military sample (Shortland et al., 2020). Taken together, and in extension to the wealth of general research on maximization, despite the small sample size here, a body of evidence is building which shows that maximization impacts decision-making in applied, high-uncertainty settings. From a theoretical sense, those same maximizers who struggle to pick phone plans, may also struggle with decisions under pressure when it really counts. While such bold assertions require significant future research, this paper, along with those before it (Shortland et al., 2020) at the very least show the importance of maximization outside of “the lab.”

Another potential limitation is the inclusion of military scenarios in the LUCIFER battery. While these scenarios deviate the user from situations that they are familiar with, or potentially trained on, this is an important benefit of LUCIFER in that it allows a test of effect across both domain-specific and domain-general scenarios. For example, it is possible that in the domain-general decisions (in this instance, police decisions), the decision-maker could be incorporating wider exogenous and endogenous concerns such as accountability and police blame-culture (Alison et al., 2013a). In this study then the inclusion of domain-general decisions allowed us to focus on the role of maximization *writ large*. That said, it is evident that varying scenario types does, de facto, increase the diversity of the decisions that were made. In response to this, we would encourage future research to better adopt a research methodology that is able to closely match the decision being made to the types of decision that the decision-maker frequently makes. This would allow future explorations pertaining to the interaction of trait maximization with experience, training and expertise.

### Implications

The National Institute of Justice Strategic Research Plan for Policing (2017–2022) specifies the need to identify the factors that inform police decision-making. Here we have identified one such factor that may play a central role in police decision-making. While likely one of many relevant personality variables that will predict individual differences in decision-making, maximization may be particularly relevant. Pais and Felgueiras (2016) work showed that time pressure and incomplete knowledge increased decision makers’ tendency to satisfice, to achieve an acceptable solution for traffic control and monitoring management. Again, this emphasizes the potential utility of satisficing in a police context. Thus, while previous research has explored the processes through which police officers overcome uncertainty (van den Heuvel et al., 2014), what this work adds is the potential role of individual decision-making traits may have on *how* individuals overcome uncertainty and commit to courses of action. The implications of this line of research should not be minimized. Increasing at the academic, political, and societal level, we are embroiled in discussions about the nature of modern policing, fueled by the outcomes of several high-profile decisions that have been made in the field. A few recent examples of which include the handling of George Floyd, the shooting of Breonna Taylor, the killing of Ahmaud Arbery, and the policing of the ensuing “Black Lives Matter” protests. One of the central discussions has been around police officers’ use of power and the decisions that they make when interacting with the public. These discussions include calls for increased officer training or more officer education (see Rydberg and Terrill, 2010), and more recently to “defund” the police (e.g., Hegarty, 2020). This paper supports that an alternate area of focus should be the matching of person to task (here, this would represent personality in terms of maximization, and experience in terms of potential service with the armed forces). This matching of person to task is a central aspect of the wider field of industrial/organizational psychology and in other high-uncertainty roles (e.g., the military), recruitment, selection, training and promotions all incorporate elements of personality (Matthews, 2013). Based on the results of this study, we could cautiously hypothesize that individuals who are maximizers may be less suited for roles that require high-uncertainty least-worst decisions as they will, by their nature, require the individual to forego maximization in favor of a satisfactory choice. As such, we would argue that focusing police organizations around the role of personality should be considered alongside calls for focusing on training and education (Rydberg and Terrill, 2010).

## Data Availability Statement

The raw data supporting the conclusions of this article will be made available by the authors, without undue reservation, to any qualified researcher.

## Ethics Statement

The studies involving human participants were reviewed and approved by the University of Massachusetts Institutional Review Board and the Army Research Institute Foundational Science Research Unit. Written informed consent for participation was not required for these studies in accordance with national legislation and institutional requirements.

## Author Contributions

NS: conceptualization, methodology, data curation, and writing original draft. LT: writing original draft, data curation, and formal analysis. LA: conceptualization, methodology, supervision, and review. All authors contributed to the article and approved the submitted version.

## Conflict of Interest

The authors declare that the research was conducted in the absence of any commercial or financial relationships that could be construed as a potential conflict of interest.
